# The Impact of Vanadium Oxide Cocatalysts on the Photocatalytic Performance of Strontium Titanates

**DOI:** 10.3390/ijms27114889

**Published:** 2026-05-28

**Authors:** Lilla Szalma, Árpád Turcsányi, Kadosa Sajdik, Karolina Solymos, Csaba Bús, Szabolcs Kocsis Szürke, Ákos Kukovecz, Zoltán Kónya, Zsolt Pap, Áron Ágoston

**Affiliations:** 1 Department of Applied and Environmental Chemistry, University of Szeged, Rerrich Béla Square. 1, H-6720 Szeged, Hungary; szalma.lilla.k@gmail.com (L.S.); kakos@chem.u-szeged.hu (Á.K.); konya@chem.u-szeged.hu (Z.K.); 2 MTA-SZTE Lendület “Momentum” Noble Metal Nanostructures Research Group, University of Szeged, Rerrich B. Square 1, H-6720 Szeged, Hungary; tarpad@chem.u-szeged.hu; 3 Interdisciplinary Excellence Center, Department of Physical Chemistry and Materials Science, University of Szeged, Rerrich B. Square 1, H-6720 Szeged, Hungary; 4 Department of Physical Chemistry and Materials Sciences, University of Szeged, Aradi vértanúk Square 1, H-6720 Szeged, Hungary; sajdik.kadosa@chem.u-szeged.hu; 5 Department of Physical and Environmental Geography, University of Szeged, Egyetem Street 2–6, H-6722 Szeged, Hungary; solymoskarolina@geo.u-szeged.hu; 6 Department of Molecular and Analytical Chemistry, University of Szeged, H-6720 Dóm Square 7–8, 6720 Szeged, Hungary; bcsaba0312@gmail.com; 7 Central Campus Győr, Széchenyi István University, Egyetem Square 1. 303., H-9026 Győr, Hungary; kocsis.szabolcs@ga.sze.hu; 8 Centre 3B, Laboratory of Advance Hydrobiology and Biomonitoring, Babeş-Bolyai University, Clinicilor 5–7, R-400294 Cluj-Napoca, Romania; 9 Hungarian Department of Biology and Ecology, Faculty of Biology and Geology, Babeş-Bolyai University, Republicii 44, R-400294 Cluj-Napoca, Romania

**Keywords:** strontium titanate, vanadium pentoxide, tetravanadium nonoxide, photocatalysis, phenol conversion, electron trapping

## Abstract

The photocatalytic activity of semiconductors can be tuned by changing their morphological or structural properties. However, a simpler and direct method is the introduction of a cocatalyst, for example V_2_O_5_ or V_2_O_5_/V_4_O_9_. In the present work, this was the cocatalyst added to SrTiO_3_. The deposition method was directed in such a way that the cocatalyst did not cover the surface of the SrTiO_3_ completely. This way, the photocatalytic process (phenol conversion) takes place at the surface of the main catalyst, while the lifetime of the generated charge carriers is increased through electron trapping via the presence of vanadium oxides. The V_2_O_5_/V_4_O_9_ cocatalyst influences the recombination processes of excited electrons in SrTiO_3_ by modifying the near-surface defects of SrTiO_3_, and it can efficiently capture electrons due to the formed heterojunction. The V_4_O_9_ content enables efficient electron transfer, as its structure can accommodate V^4+^ in addition to V^5+^. Therefore, a mixed-phase semiconductor is more suitable as a cocatalyst than a single-phase semiconductor. In this work, the photocatalytic activity of SrTiO_3_ was investigated in the presence of V_2_O_5_ (0–20 wt.%). It was found that all the samples that contained the cocatalyst showed higher photocatalytic activity than the unmodified SrTiO_3_. The sample containing 10 wt.% of cocatalyst performed ~5.4 times better than pristine SrTiO_3_ (35.87 µmol_phenol_/g_catalyst_, vs. 7.74 µmol_phenol_/g_catalyst_). This sample also contains a relatively high amount of V_4_O_9_ compared to the other samples, in addition to V_2_O_5_, which may be the main reason for the enhanced photocatalytic performance.

## 1. Introduction

To address the environmental problems of the near future, heterogeneous photocatalysis is an emerging process that could provide solutions in the fields of water and air purification, among others [1,2,3,4]. The method involves the use of semiconductor particles that are activated by a specific electromagnetic radiation level equal to or higher than their band gap [5,6]. Strontium titanate (SrTiO_3_) is a perovskite-type semiconductor with a wide, indirect bandgap (3.25 eV). This material possesses several attractive properties, such as photochemical stability and a suitable band structure [7,8]. Due to these beneficial properties, SrTiO_3_ is increasingly popular in energy storage, fuel cell, sensor, and photocatalytic applications [9,10,11].

Its conduction band edge is more negative than that of TiO_2_ at ≈200 mV, which offers favorable energetics for photocatalytic water splitting or CO_2_ reduction [12,13]. However, its indirect bandgap is 3.25 eV, which limits its photocatalytic and other light-activated applications to the ultraviolet (UV-A) range. There are several ways to improve this: changing the band gap (structural change) via doping or forming composites (metal deposition, other photocatalysts or cocatalysts), which result in different types of heterojunctions [14,15,16,17]. In the case of doping, a foreign substance is incorporated into the lattice, creating a new energy level within the SrTiO_3_ band gap. This results in a new electron band that, under favorable conditions, falls within the visible light spectrum. Another method involves manipulating the quality and quantity of defects. If the structure is enriched with a defect whose bound-electron transition energy lies within the band gap, the same effect can be achieved as with the new energy level created by doping. A further option is incorporation into a composite, for example by introducing a plasmonic noble metal, allowing the sample to absorb visible light through the utilization of LSPR, or by creating heterojunctions. In the case of SrTiO_3_, the most common configurations are Type I, Type II, and Z-scheme heterojunctions. In the Type I (straddling) configuration, the bandgap of the cocatalyst lies within the bandgap of SrTiO_3_ (Figure S1), so both charge carriers are transferred to the cocatalyst, because this is energetically favored. However, recombination is highly likely, since the charge carriers accumulate in the same region. An example of such a system is SrTiO_3_/CZTS/Ag (SrTiO_3_/copper-zinc-telluride-sulfide/silver) [18]. In the Type II (staggered) configuration, the cocatalyst’s conduction band (CB) potential is lower than that of SrTiO_3_, and the same relationship applies to the valence band (VB) (Figure S1). In this case, the excited electron of SrTiO_3_ is transferred to the cocatalyst’s CB, and the holes can theoretically be transferred from the cocatalyst to the SrTiO_3_ VB. However, this latter process is slower than electron transfer. An example of such a system is SrTiO_3_/Ag_2_O [19]. In the Z-scheme configuration, the cocatalyst’s CB has a higher potential than the SrTiO_3_ CB (Figure S1). The excited electron in SrTiO_3_ transfers from its CB to the cocatalyst’s VB, quenching the hole on the cocatalyst. As a result, the charge carriers with the highest energy remain: the excited electron in the cocatalyst’s CB and the hole in the SrTiO_3_ VB. An example of such a system is g-C_3_N_4_/SrTiO_3_ [20]. Other methods include changing the morphology (specific surface area and anisotropy) of the particles or modifying the crystal structure [21,22].

Vanadium and vanadium oxides are in focus for different applications, due to their multiple possible oxidation states, which can vary between +5 and +2. This versatility leads to a large spectrum of materials with different physicochemical (electrical, optical, optoelectronic, and magnetic) properties [23]. Composites containing V_2_O_5_ can be beneficial in various applications, such as solar cells as hole transport materials, batteries as cathodes, gas sensors, catalysis, and photocatalysis [24,25,26,27]. Vanadium pentoxide (V_2_O_5_) is thermodynamically the most stable among the vanadium oxides [23,28]. V_2_O_5_ is a poor stand-alone photocatalyst, but it can be used effectively as a cocatalyst. The band gap of V_2_O_5_ varies widely depending on its structure and the measurement technique used, ranging from 2.2 to 2.8 eV (443–564 nm) within the visible light energy range [29]. This work also involves V_4_O_9_. This is a metastable oxide capable of transforming completely into V_2_O_5_; however, the process requires energy, so it occurs at high temperatures and in an oxidative atmosphere. V_4_O_9_ is a mixed oxide: the vanadium component exists as both V^4+^ and V^5+^ species. In a published study, the band gap of V_4_O_9_ synthesized using the ultrasonic spray pyrolysis technique falls within the visible light range, at ~2.3–2.4 eV [30]. What makes its potential use as a cocatalyst promising is that its structure stably contains V^4+^ and V^5+^. The trapping of the photocatalyst’s excited electron (e.g., V^5+^ ⇆ V^4+^) can therefore persist for a longer time, allowing the remaining positive hole on the photocatalyst to be utilized more efficiently.

Since vanadium can easily change its oxidation state, it has the potential to be an efficient and reversible electron trap. Therefore, it is suitable for increasing the photocatalytic efficiency of commonly used photocatalysts (TiO_2_, ZnO, Al_2_O_3_, WO_3_) through the formation of binary composites. Su et al. prepared V_2_O_5_–BiVO_4_ composites with heterojunctions, leading to a significant increase in photocatalytic performance, through an increase in the exciton separation rate and the lifetime of photogenerated charge carriers [31]. Similarly, the increased photocatalytic performance of MoS_2_–V_2_O_5_,V_2_O_5_/RGO, V_2_O_5_/TiO_2_, and ZnO/V_2_O_5_ photocatalysts can be attributed to increased light absorption capacity, efficient charge transfer, and minimal charge recombination [32,33,34].

In this work, V_2_O_5_/V_4_O_9_ containing strontium titanate composite photocatalysts were synthesized to investigate the composition dependence of the UV-A-driven photocatalytic activity of SrTiO_3_. It was observed that the presence of V_4_O_9_ generated along with V_2_O_5_ had a positive effect on photoactivity. There are no reports in the current literature that investigate the influence of V_2_O_5_ or V_4_O_9_ on the photocatalytic performance of SrTiO_3_. Although a few studies examine these materials in combination, their focus is limited to dielectric properties rather than photocatalytic behavior [35,36].

## 2. Results

### 2.1. Characterization

**Figure 1:**
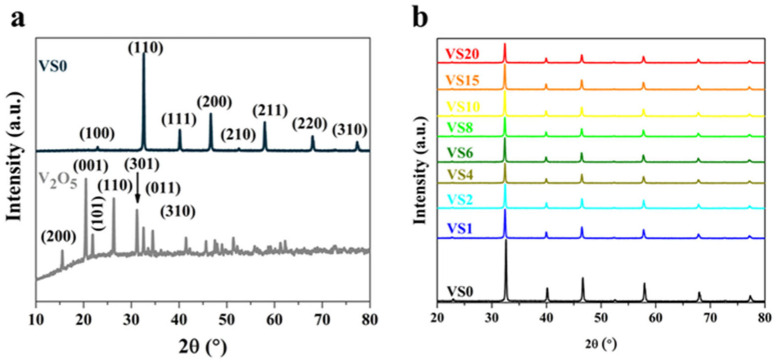
The XRD patterns of pristine SrTiO_3_, V_2_O_5_ (**a**), and VS composite samples (**b**).

During the structural analysis (Figure 1a), cubic SrTiO_3_ (VS0) (JCPDS No. 35-0734) was identified, with signals corresponding to the (100), (110), (111), (200), (210), (211), (220), and (310) crystallographic planes. In the case of V_2_O_5_, the orthorhombic crystal phase was found, with characteristic diffraction peaks of (200), (001), (101), (110), and (301) (JCPDS No. 41-1426). In the diffraction pattern of the composite VS samples, just the presence of SrTiO_3_ was detected. This suggests that the amount of crystalline V_2_O_5_ or other vanadium containing oxide, which is certainly present, is too low, or that all of them are amorphous. This issue will be clarified in the later stage of the manuscript, when other characterization techniques are discussed. The presence of any form of V_2_O_5_ did not influence the crystal structure of SrTiO_3_, as the diffraction patterns of the samples did not change following the deposition of vanadium species.

**Figure 2:**
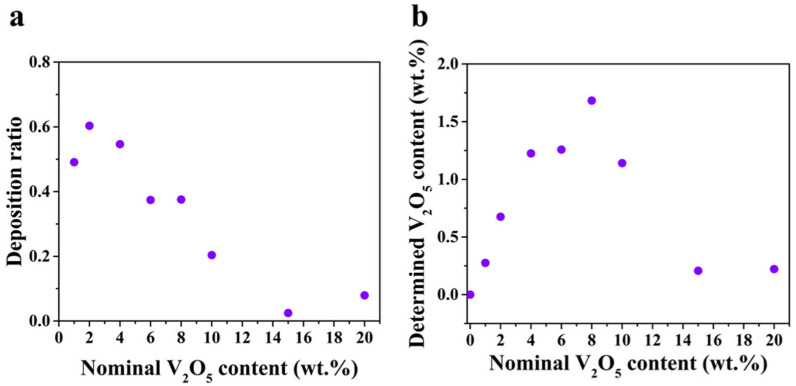
Deposition efficiency (**a**) and the determined real vanadium content (**b**).

The real vanadium content (please note that the section discussing the X-ray fluorescence spectroscopy (XRF) results will refer to vanadium content which is in direct relationship with the oxide amount in the samples) and the deposition efficiency were calculated using XRF measurements (Table 1). It was found that the real vanadium content of the sample was below the nominal values (Figure 2), as expected. The highest deposition ratio of vanadium was achieved at low nominal vanadium concentrations. The efficiency decreased as the nominal concentration increased (Figure 2a). There may be several reasons for this.

One reason may be that the higher vanadium precursor concentration resulted in more crystal nuclei in the solution phase compared to the surface of SrTiO_3_. Hence, those particles that were not attached to the surface of SrTiO_3_ were washed out after crystallization [37]. A possible reason may be that larger particles were formed, which are less able to cover the SrTiO_3_ surface for steric reasons, and the residue is also washed out in this case. A further explanation could be that, as the concentration of the surfactant and the added vanadium source increased, more and/or larger micelles were formed [38], which stabilize and mobilize the newly formed vanadium oxide particles, preventing the formation of junctions between vanadium species and the SrTiO_3_ surface. Another possible explanation is that, since NH_4_Cl reduces the pH to between 5 and 6, where SrTiO_3_ has its isoelectric point, this reduces the amount of negatively charged vanadate bound to the surface. Synthetic variants containing nominally more vanadate contain more NH_4_Cl, thus enhancing this effect [39]. To confirm the presence of vanadium-containing oxide on the surface, SEM mapping and EDX analysis were performed on the sample with the highest vanadium content (VS8). The results are presented in the Supplementary Material (Figure S2) and confirm the findings from the XRF analysis.

To determine the specific surface area of the samples, complete N_2_ adsorption–desorption isotherms were recorded; a summary figure for the samples is shown in Figure S3. The specific surface areas were calculated according to the BET method based on the initial linear segment, and the resulting values are listed in Table 1. Commercial SrTiO_3_ (VS0) has a very low specific surface area, which was only slightly increased by modifying the sample with V_2_O_5_ and V_4_O_9_. This indicates that the surface of commercial SrTiO_3_ is relatively smooth and uniform, exhibits low porosity, and contains low-level anisotropy. The deposition of the cocatalyst therefore results a minimal increase in the specific surface area.

**Figure 3:**
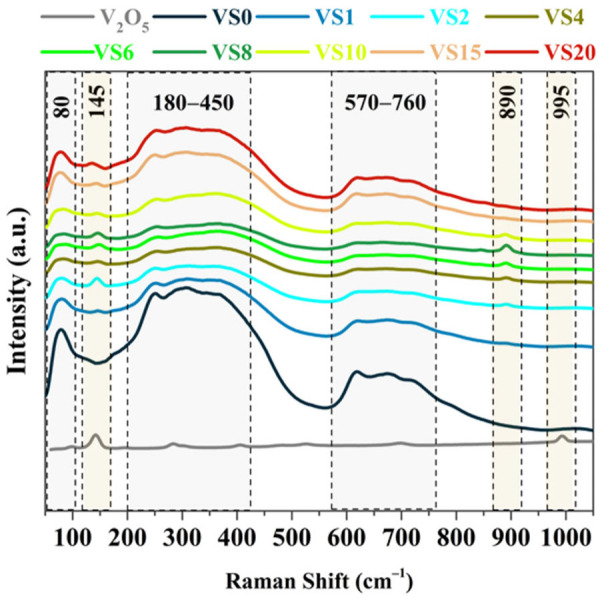
The Raman spectra of the vanadium-modified SrTiO_3_ samples.

Raman spectroscopy was employed to identify the different vanadium-containing species formed on the surface of SrTiO_3_ (Figure 3). The region spanning 180–450 cm^−1^ and 570–760 cm^−1^ is related to the second-order Raman active mode of SrTiO_3_ [40,41,42]. The peak at 80 cm^−1^ cannot be attributed to a specific bond; it corresponds to the rotation of the [TiO_6_]^8−^ octahedral [43]. At 145 cm^−1^, the V–O–V bond was detected in all modified samples (a signal specific to V_2_O_5_). The only exception was sample VS1, most likely due to the low vanadium concentration. This signal indicates that the synthesis method mainly resulted in V_2_O_5_ [44,45,46]. A supporting signal for the presence of V_2_O_5_ could be the one located at 995 cm^−1^ (V=O stretching bond from V_2_O_5_); however, it was detectable only in the pristine V_2_O_5_ [45,46].

At 890 cm^−1^, a V=O signal was detected which can be assigned to the presence of V_4_O_9_, an oxide containing both V^4+^ and V^5+^. The former species can be obtained if a slightly reductive atmosphere is present when V_2_O_5_ is exposed to heat or if the medium is not oxidative enough [46]. This signal is not visible in VS1, but appears in VS2 and increases in intensity with the nominal V_2_O_5_ concentration. If the spectra are normalized, the most intense V_4_O_9_ signal was found in the VS8 sample, meaning that the largest amount of V_4_O_9_ is present here. This trend closely follows the actual vanadium content (XRF) in the composite: the more vanadium was detected in the composite, the more V_4_O_9_ is detected. All these observations suggest that the deposition of V_2_O_5_ from the precursor (going through several intermediate formation steps, such as V_4_O_9_) is not only oxygen- and heat-intensive, but also slow. Hence, this procedure requires longer calcination times, making the calcination parameters the key factors. During the XRD analysis, V_4_O_9_—similarly to V_2_O_5_—was not detected, most likely because its amount is below the detection limit or it is present in an amorphous form. For each vanadium-containing sample, the integrated area under the peaks at 145 cm^−1^ and 890 cm^−1^ was determined. The figures (Figures S4 and S5) and area ratios (Table S1) are provided in the Supplementary Material. The determined ratio is not equal to the V^4+^/V^5+^ ratio, from which we could estimate the V_4_O_9_ content; the determined ratio only allows for relative comparison. Figures S4 and S5 also show that the 145 cm^−1^ and 890 cm^−1^ signals are detectable in all modified samples, indicating that V_4_O_9_ is present in all of them.

**Figure 4:**
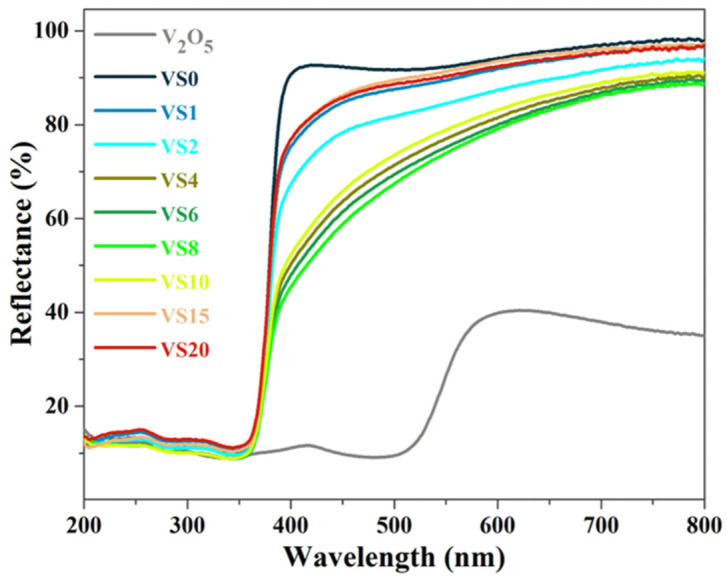
Diffuse reflectance spectra of the VS sample series.

The diffuse reflectance spectrum of the samples is shown in Figure 4. The band gap value was ~3.26 eV in all cases, as determined by taking the first-order derivative of the reflection spectra (Figure S6). This indicates that the structure of SrTiO_3_ has not been altered via doping or any other crystallization-related phenomena [47]. By increasing the nominal V_2_O_5_ content, the absorbance in the visible range increases accordingly until sample VS8, after which it decreases again. The reason for this is that this sample contains the highest amount of V_4_O_9_ (according to Raman measurements), which absorbs a higher amount of visible light compared to V_2_O_5_ [48]. These results are in line with the ones obtained from the XRF and Raman spectroscopy measurements.

**Figure 5:**
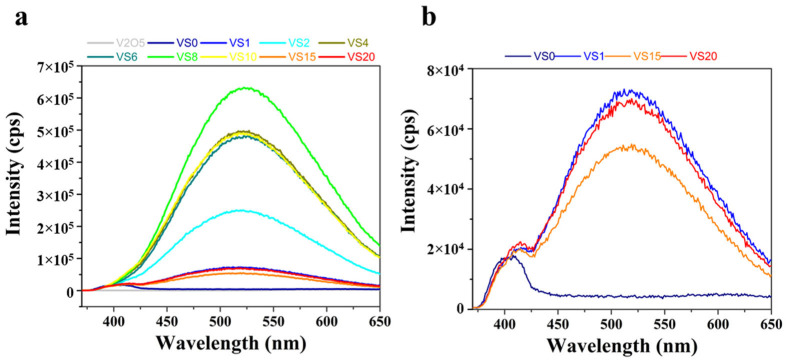
Photoluminescence spectra of the VS samples (**a**) and the magnification of low-emitting samples (**b**).

The photoluminescence measurements (PL) showed two emission bands: one less intense band located at 410 nm and a more intense one at 520 nm (in the VS samples), as shown in Figure 5. The 410 nm emission is a near band-edge emission (NBE) close to the band gap (380 nm), which occurs in SrTiO_3_ samples measured at low temperatures and can be attributed to phonon-assisted transitions between two energy states [49]. The electron transition associated with NBE emission is most clearly visible in those samples where it is not overlapped by the emission at the 520 nm level: these are VS0, VS1, VS15, and VS20 (for better visibility, only the PL spectra of these four samples are shown in Figure 5b). The green emission detected at 520 nm is the radiative recombination of self-trapped excitons generated during the excitation of SrTiO_3_ (self-trapping may occur at this emission due to Ti^3+^ defect sites) [50]. XRF measured the lowest vanadium content in the case of VS0, VS1, VS15, and VS20, so its effect on the surface defects of SrTiO_3_ is the smallest in these cases. This effect causes the passivation that results in the emphasis of radiative emission from the level associated with the 520 nm electron transition. As SrTiO_3_ was already synthesized, and the V_2_O_5_ deposition procedure followed after this, any change in the PL spectra can be directly linked to the electron transfer processes between the two materials, lattice rearrangement of SrTiO_3_ caused by calcination, and the effect of the foreign material (V_2_O_5_/V_4_O_9_) on near-surface defects. Since the increase in intensity associated with the 520 nm emission appears in those samples where Raman measurements detected the presence of V_4_O_9_, this enhancement can be attributed to the effect of V_4_O_9_. The underlying mechanism is that the V_2_O_5_/V_4_O_9_ cocatalyst containing V_4_O_9_ at the interface electrostatically affects SrTiO_3_ surface defects, thereby passivating the “competitive” levels associated with the 520 nm band and inhibiting thermal recombination. Therefore, more excited electrons are transferred to the energy level associated with the 520 nm transition, where radiative recombination is favored. The intensity of the photoluminescence increases with the nominal concentration of V_2_O_5_, reaching a maximum value in the case of sample VS8 (which had the highest V_2_O_5_/V_4_O_9_ content), and then decreases again. The same trend was observed in the DRS and XRF measurements, where the maximum value was reached in the same sample. Thus, the presence and amount of V_2_O_5_ and/or V_4_O_9_ influence the effect of the defects in strontium titanate.

**Figure 6:**
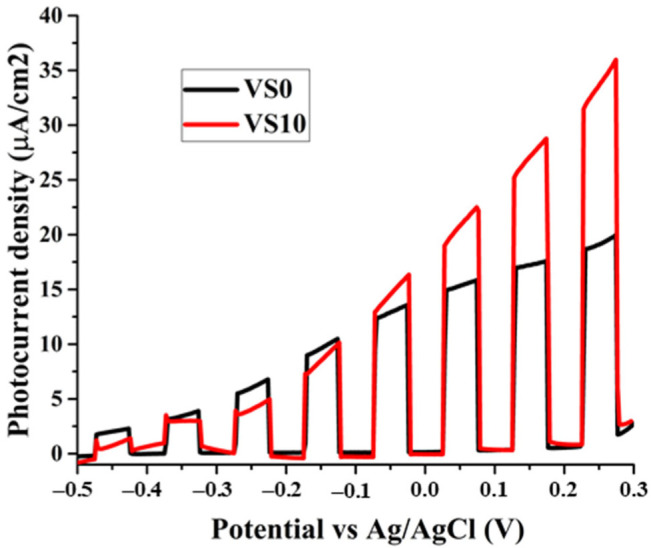
Photocurrent density of the modified SrTiO_3_ (VS10) and the pristine SrTiO_3_ (VS0).

The photocurrent density was measured for sample VS10, which was the best performing photocatalyst, alongside the VS0 reference sample (Figure 6). The former showed a higher photocurrent density compared to VS0. In n-type semiconductors (e.g., SrTiO_3_), photogenerated electrons can only move toward the electrode due to band bending, but holes can be utilized on the surface in contact with the solution, where they can oxidize suitable electron donor components [51]. V_2_O_5_ and V_4_O_9_ may reduce the amount of photocatalytically active sites on SrTiO_3_ in sample VS10; however, the cocatalyst can trap the photogenerated electrons. If the positive effect of the latter is greater than the negative effect of the former, a higher photocurrent can be measured. Due to the electron trapping effect, the lifetime of the photogenerated holes increases; thus, the possibility of reaction with the donor (sulfite anion in our case) increases. Since higher photocurrent was measured for the modified SrTiO_3_ than for the unmodified, pristine SrTiO_3_, the electron trapping effect of V_2_O_5_ and V_4_O_9_ was confirmed. The charge transfer scenarios suggest the presence of photogating, during which an enhanced photoresponse is obtained in the form of a current [52,53]. Electron trapping is facilitated by the fact that, at the interface between two semiconductors with different structures (heterojunction), band bending occurs due to the differing band structures. Generally, the CB bands of the two materials bend toward each other, thereby reducing the energy gap between the two CB bands and facilitating electron transfer.

### 2.2. Photocatalytic Activity

**Figure 7:**
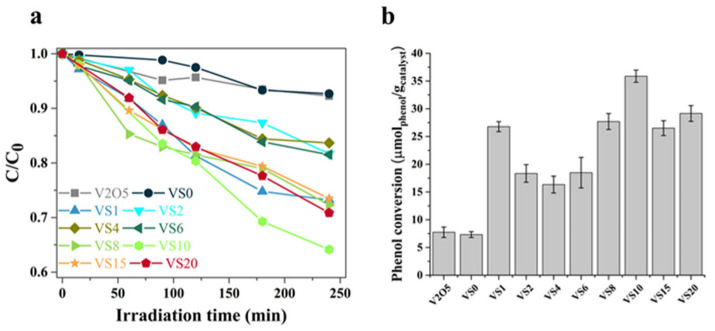
The photodegradation curves of phenol (**a**) and the conversion rate values (**b**).

The photocatalytic efficiency of the samples was measured through the photocatalytic conversion of phenol (Figure 7a,b). Each sample was tested repeatedly, and the results are presented in the Supplementary Material (Figures S7 and S8). Phenol adsorption is negligible on both SrTiO_3_ and the modified samples. This is explained by their extremely low specific surface area (1.43–3.90 m^2^∙g^−1^) and by numerous publications reporting that adsorption on SrTiO_3_ and other photocatalysts does not occur or occurs only to a minimal extent (~1%) [54,55,56].

In the case of the SrTiO_3_-V_2_O_5_/V_4_O_9_ system we designed, based on the results of material characterization (PL, XRF, Raman), the cocatalyst can enhance photocatalytic efficiency in two ways. In one case, the excited electron is transferred via the heterojunction from the SrTiO_3_ conduction band to the V_2_O_5_/V_4_O_9_ conduction band, where it can be trapped or recombine at a defect site. This statistically increases the lifetime of charge carriers, so that the positive holes remaining on SrTiO_3_ induce oxidation reactions on the surface at a higher rate than without a cocatalyst, when recombination processes are more dominant. The electron transfer from the photocatalyst to the cocatalyst is energetically favored from the beginning, but the resulting Schottky junction further enhances successful electron migration. The Schottky junction is the phenomenon where the bands bend at the interface between a semiconductor and another semiconductor/metal, as the differing band structures exert an electrostatic influence on each other. The result is that the bands of the two materials through which electron migration occurs become energetically closer to each other.

Another reason we identified during the PL measurements is that the V_2_O_5_/V_4_O_9_ cocatalyst on the SrTiO_3_ surface affects the near-surface defects in SrTiO_3_, which play a role in the recombination processes of excited electrons. Since the intensity increased from a level associated with one of the defect sites, this indicates that a trap site was passivated, where the electron would have recombined non-radiatively in the absence of the cocatalyst. This passivation is important because, since the electron did not recombine at the passivated defect site in SrTiO_3_, but instead went to the energy level associated with the 520 nm recombination, the V_2_O_5_/V_4_O_9_ cocatalyst has a higher probability of trapping it. This is statistically possible because radiative recombination processes are significantly slower than non-radiative recombination processes.

The modified samples showed higher photocatalytic activity than unmodified SrTiO_3_. The deposition of vanadium oxides significantly improved the photocatalytic activity (VS1 sample), which then decreased continuously until sample VS4, after which it started to increase again until sample VS10, and then decreased again. The reason for the initial decrease is probably that, although electron trapping occurs on the cocatalyst, the cocatalyst does not conduct the electrons sufficiently, since it is not available in sufficient quantities (VS1–VS4), allowing them to recombine after a certain period of time [57]. Another reason is that the cocatalyst increasingly covers the free surface of SrTiO_3_, reducing the number of photocatalytic sites and also exerting a shading effect. Later, when the photocatalytic activity increases (VS4–VS10), there is already a sufficient amount of V_2_O_5_/V_4_O_9_ cocatalyst present, so charge separation improves, and the remaining positive holes can exert their effect more effectively. These results indicate that the V_2_O_5_/V_4_O_9_ mixed oxide enhances charge separation more effectively than V_2_O_5_, suggesting that it serves as a more efficient electron trap.

The sample VS10 showed the highest photocatalytic activity due to the optimal amount of V_2_O_5_/V_4_O_9_ on the SrTiO_3_ surface, which is less than in most samples (XRF). Therefore, few active sites are covered on the surface of SrTiO_3_ where the reaction takes place, but there are still enough V_2_O_5_ and V_4_O_9_ particles present to manifest their electron-trapping role [58]. V_4_O_9_ has a greater beneficial effect on electron trapping: V^5+^ can easily accept an electron and form V^4+^, because its structure is capable of accommodating V^4+^ (since it is already present). It can therefore be concluded that the photocatalyst containing a mixed oxide effectively enhances photocatalytic activity.

## 3. Materials and Methods

### 3.1. Materials

Strontium titanate (SrTiO_3_, >99%, Alfa Aesar, Haverhill, MA, USA), sodium metavanadate (NaVO_3_, >98%, Sigma-Aldrich, St. Louis, MO, USA), ammonium chloride (NH_4_Cl, reagent grade, Vwr International Kft., Debrecen, Hungary), cetyl trimethyl ammonium bromide (CH_3_(CH_2_)_15_N(Br)(CH_3_)_3_, CTAB, >98%, Sigma-Aldrich, St. Louis, MO, USA), ultrapure Milli-Q water (from a Millipore Milli-Q cleaning system, Merck Millipore, Burlington, MA, USA), and vanadium (V) oxide (V_2_O_5_, >99%, Acros, Geel, Belgium) were used during the experiments without further purification.

### 3.2. Synthesis of V_2_O_5_- and SrTiO_3_-Containing Cocatalysts

SrTiO_3_-based composites with different V_2_O_5_/SrTiO_3_ mass ratios (0–20 wt.% of nominal V_2_O_5_) were fabricated based on a synthesis procedure available in the literature [59]. Before the first step, a sodium metavanadate solution (0.25 mol·L^−1^), an ammonium chloride solution (3 mol·L^−1^), and a CTAB solution (0.2 mol·L^−1^) were prepared. A suspension of SrTiO_3_ was prepared in the desired ratio by sonication for 10 min, and the mixture was kept in suspension by stirring at 4 rpm. The suspensions were prepared using a volume of water such that the final volume was 200 mL after all reagents had been added. First, CTAB was added to the SrTiO_3_ suspension (n_CTAB_/n_V2O5_ = 2/1). Then, while stirring continuously, the system was heated to 60 °C. Next, a NaVO_3_ precursor solution corresponding to the desired V_2_O_5_ content was added. Following this, NH_4_Cl was added (n_NaVO3_/n_NH4Cl_ = 1/2), and the mixture was stirred vigorously at 60 °C for 3 h in a total volume of 60 mL. Next, the product was collected, washed four times with Milli-Q water (centrifugation for 5 min at 4400 rpm), dried at 40 °C for 12 h in air, and ground in an agate mortar. The product was then calcined in a tube furnace using a continuous O_2_ flow for 4 h at 600 °C, with a 10 °C·min^−1^ heating rate. The samples were coded as “VSX”, where “V” corresponds to V_2_O_5_, “S” to SrTiO_3_, and “X” to the nominal V_2_O_5_ content in wt.%.

### 3.3. Characterization

A Rigaku Miniflex II X-ray diffractometer (Rigaku, Neu-Isenburg, Germany) was used to determine the crystalline composition of the samples. For SrTiO_3_ and V_2_O_5_, the measurement range was 20–80 2θ°, and for the composite samples, it was 10–45 2θ°, corresponding to the region of the most intense peaks of both SrTiO_3_ and V_2_O_5_. During the measurements, the following parameters were used: λ_CuKα_ = 0.15406 nm, 30 kV, and 15 mA.

To determine the vanadium content, X-ray fluorescence measurements were carried out using an Olympos VMR X-ray fluorescence (XRF) spectrometer. With a 50 keV excitation X-ray beam, the detectable fluorescence of the vanadium Kα_1_ electron transition is 4.9 keV, which can be used to determine the vanadium content of a sample [60]. By comparing the nominal and real vanadium content, the efficiency of V_2_O_5_ deposition process can be examined on the surface of SrTiO_3._

SEM-mapping images were taken of the elements comprising the sample with a Thermo Fisher Scientific Apreo 2 instrument (Waltham, MA, USA). The microscope was operated at 25 pA current and 15 kV acceleration voltage.

The quality of the vanadium content in the samples was examined using Raman spectroscopy. The Raman spectra of the samples were recorded using a SENTERRA II Raman microscope (Bruker Optics, Inc., Billerica, MA, USA). The excitation laser wavelength was 785 nm, with an integration time of 10 s (5 repetitions), a resolution of 4 cm^−1^, and an interferometer resolution of 0.5 cm^−1^.

The specific surface areas of the samples were determined by nitrogen adsorption at 77 K using a BELCAT-A device (Microtrac Retsch GmbH, Duesseldorf, Germany). The specific surface area was calculated via the BET method.

Diffuse reflectance spectra (DRS) were recorded between 200 and 800 nm using a Shimadzu UV-3600 Plus UV–VIS–NIR spectrophotometer equipped with an integration sphere (Shimadzu Corporation, Kyoto, Japan). The first-order derivative of the spectra was used to determine the band gap value of the samples [61].

The photoluminescence (PL) of the samples was investigated using a Horiba Jobin Yvon Fluoromax-4 spectrofluorometer (Horiba, Kyoto, Japan) at a 350 nm excitation wavelength. A band-pass filter (350 nm, FWHM = 10 nm) was used to provide the monochromatic excitation source. A high-pass cut-off filter (>370 nm) was applied before the monochromator of the detector to filter out the light originating from the excitation source.

To prove the electron-trapping effect of V_2_O_5_, photocurrent density was measured using a classic three-electrode system in aqueous medium. To measure the generated photocurrent, a Metrohm Autolab PGSTAT302n potentiostat/galvanostat (Metrohm AG, Herisau, Switzerland) was used. Fluorine-doped tin oxide (FTO) containing the photocatalyst was used as the working electrode. The sample layer on the FTO plate was prepared via spray coating (1 mg·cm^−2^ sample coverage). Before the spray coating, the FTO sheets were cleaned: first with acetone, then with propanol, and finally with deionized water. A platinum wire was used as the counter electrode, while an Ag/AgCl electrode (in 3 mol·L^–1^ NaCl) was used as the reference electrode. The experiments were carried out in a 0.5 molar Na_2_SO_3_ solution, which acted as the electrolyte and a hole scavenger. The intensity of the incoming light was 100 mW·cm^2^, and the current density was obtained in mA·cm^–2^. The photocurrent density was measured using linear sweep voltammetry (LSV) in the potential range of −0.5 to 0.3 V, at a sweeping rate of 10 mV·s^–1^. The spectrum of the light source used is shown in Figure S9.

TG analysis was performed using a TA Instruments TGA Q500, (TA Instruments, New Castle, DE, USA) with a 10 °C/min heating rate, a 20–700 °C range, and an air atmosphere to verify stability (V_4_O_9_). (The results can be found in the Supplementary Material, Figure S10–S13.)

### 3.4. Photocatalytic Activity Measurements

The photocatalytic activity was followed via the photodegradation of phenol. For an experiment, 100 mL of 1 g∙L^−1^ photocatalyst suspension was prepared, which contained the model pollutant (the initial concentration of phenol was 0.1 mmol·L^−1^). The homogenization of the suspension was facilitated via sonication. The experiments were conducted in a double-walled quartz cylinder (190 mL) surrounded by six fluorescent tubes (UV-A), each with a power of 6 W (Novelite T5, λ_max_ = 365 nm, the average light intensity of one fluorescent tube is 1.266 mW∙cm^−2^, the spectrum is illustrated in Figure S14), with magnetic stirring, constant air flow (to keep the dissolved oxygen concentration constant), and water circulation (to maintain a constant temperature of 25 °C). A photocatalytic experiment was performed for 240 min, preceded by 10 min of stirring in the dark to ensure that the adsorption–desorption equilibrium was achieved. Samples were taken at specific time intervals and centrifuged for 3 min (13400 rpm). The supernatant was filtered using a Whatman filter (0.02 μm pore size) and analyzed by HPLC using a Merck-Hitachi L-7100 equipped with a Merck-Hitachi l-4250 UV–Vis detector and a Lichrospher R_p_ 18 column (Merck KGaA, Darmstadt, Germany). A 35:65 (*V*/*V*) methanol/water mixture was used as the eluent (flow rate: 0.8 mL∙min^−1^), and the absorbance values were recorded at λ = 210 nm (Figure S15).

## 4. Conclusions

Using a simple and rapid synthesis method, a SrTiO_3_-based composite photocatalyst series was developed containing V_2_O_5_/V_4_O_9_ cocatalysts. The presence of vanadium oxides and their deposition efficiency changed with the nominal vanadium content (added during synthesis). Starting with the second sample, the separated/nominal vanadium ratio decreased continuously, and, in terms of absolute separated quantity, it reached a maximum value in the VS8 sample. According to the structural analysis, the structure of SrTiO_3_ did not change during the cocatalyst deposition process. The Raman spectra demonstrated that not only V_2_O_5_, but also V_4_O_9_, are present in the samples. The amount of V_4_O_9_ seems to be dependent from the total amount of deposited vanadia species. The increasing visible light absorbance observed in the DRS spectra and the increasing emission intensity observed during PL measurements follow the same trend as the total amount of deposited vanadia species. According to PL measurements, the detached vanadium influences the recombination path of the excited electron, resulting in the detection of high-intensity photon emission at ~520 nm. It was determined during PL measurements that the deposited V_2_O_5_/V_4_O_9_ cocatalyst affects the surface defects of SrTiO_3_, as it passivates a defect site where recombination is non-radiative; this is evidenced by the increase in the 520 nm emission. Thus, the separated cocatalyst modifies the recombination path of the excited electron. Due to the Schottky junction, band bending occurs at the interface, which allows the electron to more easily transfer to the cocatalyst’s CB level, thus enhancing electron trapping. Both of these phenomena (defect passivation and electron trapping) increase the probability of photocatalytic processes. Photocurrent density measurements reinforced the electron trapping role of the V_2_O_5_/V_4_O_9_ cocatalyst. As expected, the photoactivity of the composites followed a trend, which was directly dependent on the properties discussed above. With the addition of the cocatalyst, the photocatalytic efficiency of SrTiO_3_ increased. As the amount of cocatalyst was increased, the photocatalytic efficiency first decreased, then began to increase again, reaching its maximum value in the VS10 sample. According to the photocurrent density and PL measurements, this sample effectively traps electrons, increasing the availability of the holes. These results indicate that the V_4_O_9_ mixed oxide enhances charge separation more effectively than V_2_O_5_, suggesting that it serves as a more efficient electron trap. The VS10 sample is optimal in terms of V_4_O_9_ and V_2_O_5_ content, as it contains enough electron-trapping sites, but at the same time the surface of SrTiO_3_ was still available for the photocatalytic process to proceed.

## Figures and Tables

**Table 1:** Vanadium content of the samples, as measured by XRF.

Sample	Nominal V_2_O_5_ (wt.%)	Nominal V (wt.%)	Measured V (wt.%)	Deposited V Ratio	Specific Surface Area (m^2^∙g^−1^)
VS0	0	0.00	0.00	-	1.43
VS1	1	0.56	0.17	0.30	2.37
VS2	2	1.12	0.66	0.59	3.90
VS4	4	2.24	1.21	0.54	2.94
VS6	6	3.36	1.24	0.37	2.75
VS8	8	4.48	1.64	0.37	2.18
VS10	10	5.60	1.13	0.20	2.63
VS15	15	8.40	0.14	0.02	2.59
VS20	20	11.20	0.25	0.02	3.01

## Data Availability

The original contributions presented in this study are included in the article. Further inquiries can be directed to the corresponding authors.

## Supplementary Materials

The following supporting information can be downloaded at: https://www.mdpi.com/article/10.3390/ijms27114889/s1.
