# Supplementary material for: The Impact of Vanadium Oxide Cocatalysts on the Photocatalytic Performance of Strontium Titanates
Source: Int J Mol Sci. 2026 May 28;27(11):4889. doi: 10.3390/ijms27114889 (PMC13256524; doi:10.3390/ijms27114889)
Supplement: Supplementary file 1 [file ijms-27-04889-s001.zip › ijms-4245525-supplementary.pdf]

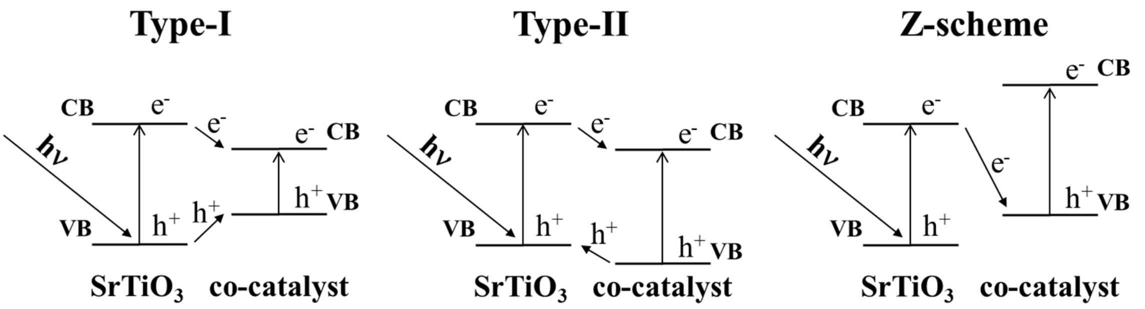

Figure S1. The most significant heterojunctions in $SrTiO_3$ composites.

The elemental map of the VS8 sample, which contains the highest amount of vanadium, is shown in **Fig. S1**. The distribution of Sr, Ti, and O is perfectly homogeneous, as expected. V_2O_5/V_4O_9 is present only on the surface of $SrTiO_3$, but since it completely covers it, a relatively homogeneous distribution is also observed for vanadium. The concentrations measured by EDX for Sr, Ti, O, and V were 48.95 wt.%, 24.42 wt.%, 24.83 wt.%, and 1.80 wt.%. In the case of XRF, 1.64 wt.% vanadium was measured; this value is higher in the EDX measurement because the information in the EDX measurement comes from a shallower depth than in XRF, and the vanadium is located on the surface.

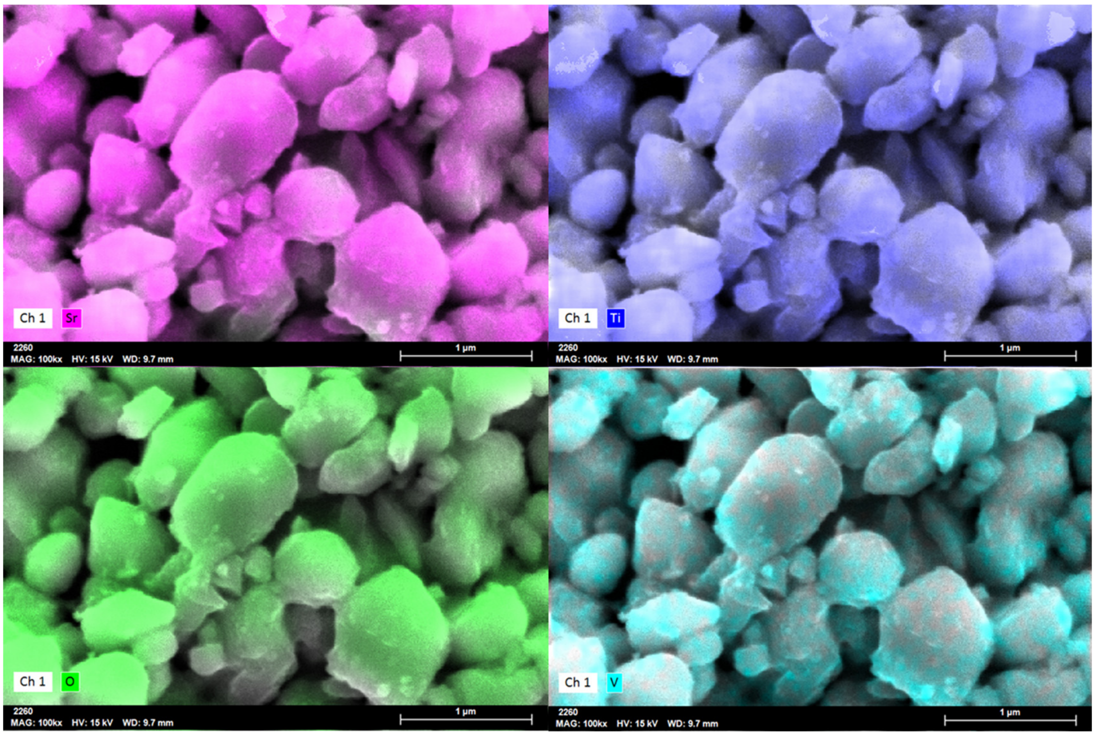

Figure S2. SEM-mapping of Sr, Ti, O and V elements for the VS8 sample .

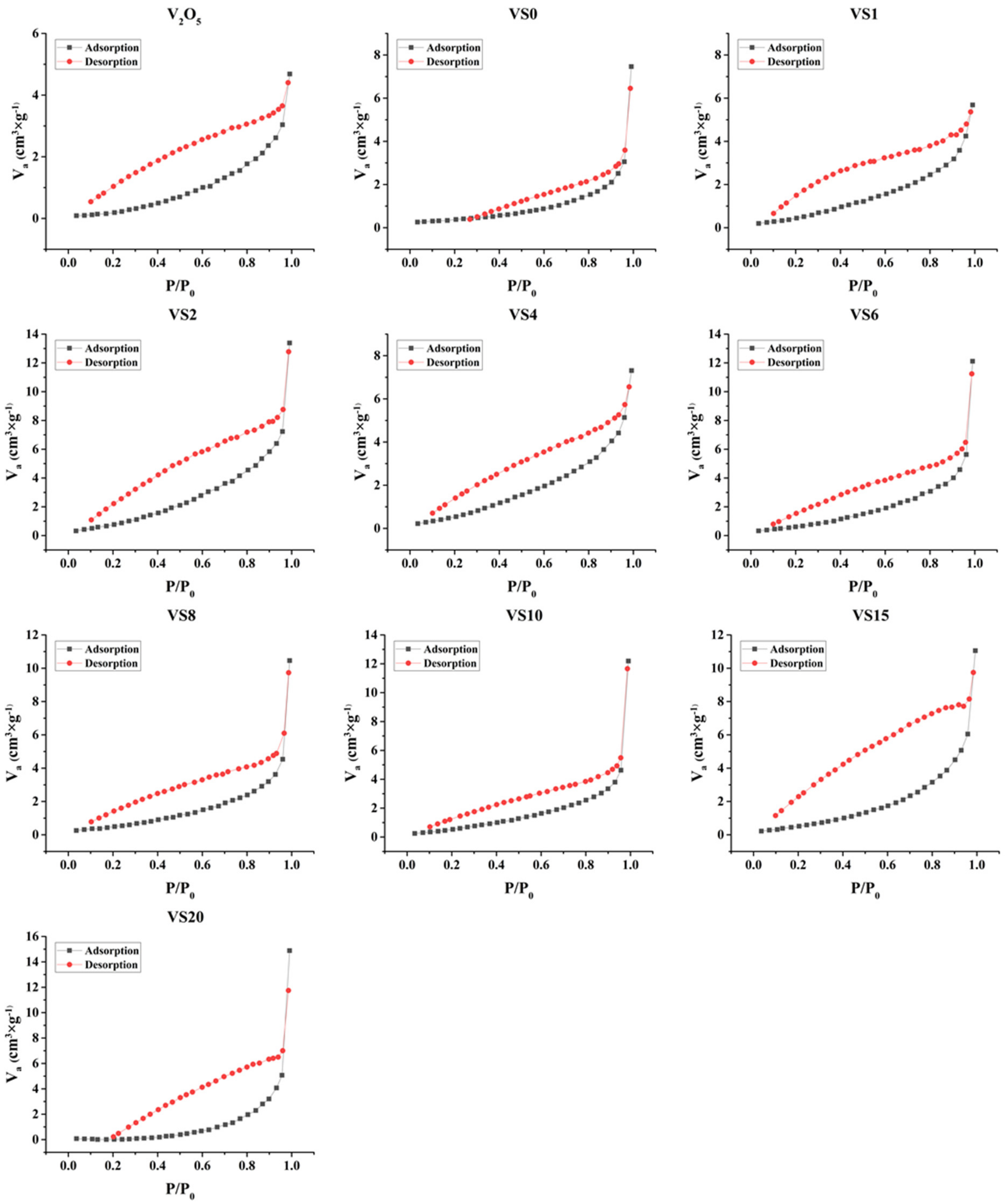

Figure S3. N_2 adsorption-desorption isotherms of the studied samples .

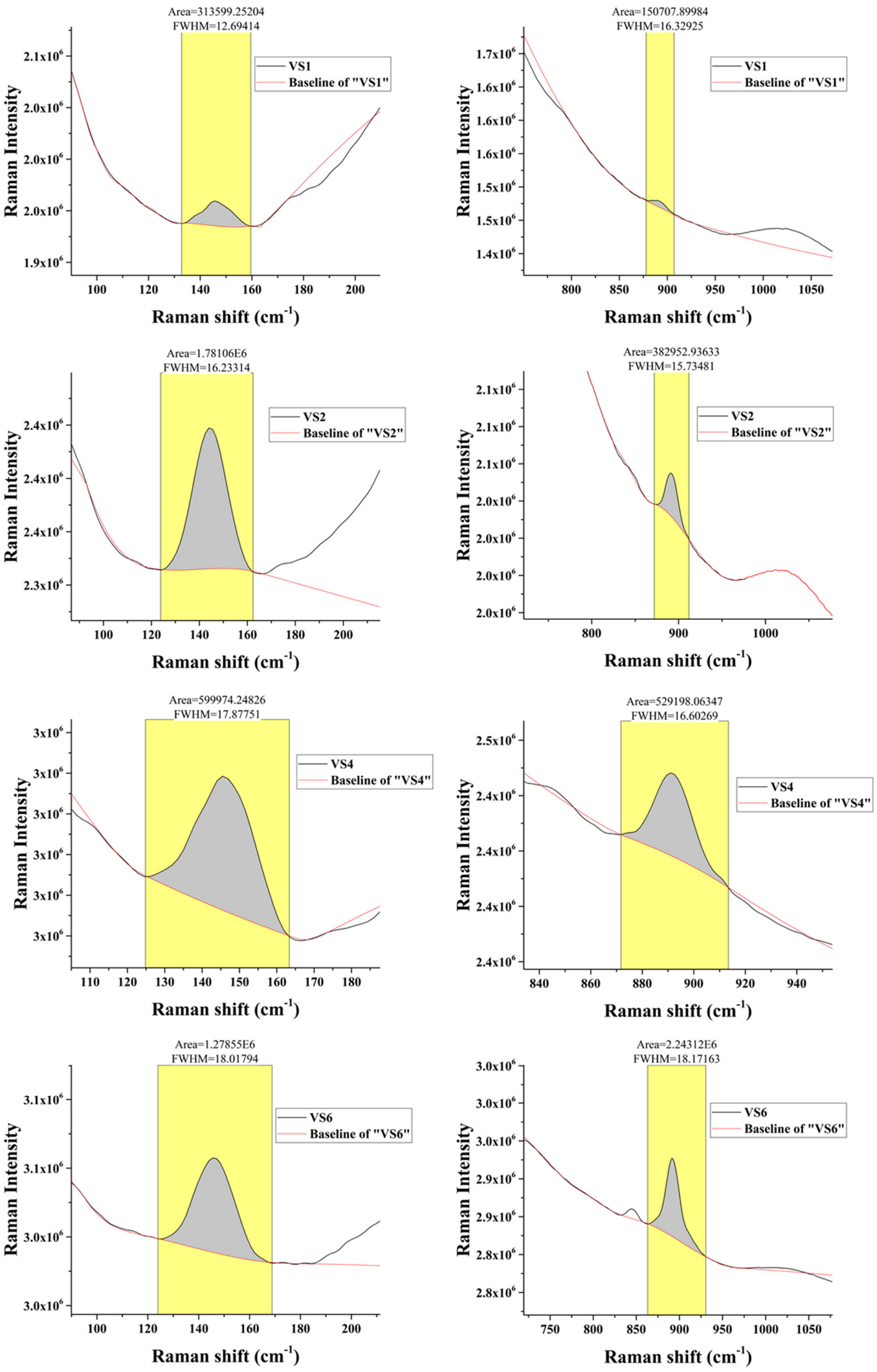

Figure S4. Detail of the Raman spectra of the studied samples. Area under the peaks at 145 and 890 cm^{-1} that corresponds to the VS1-VS6 samples.

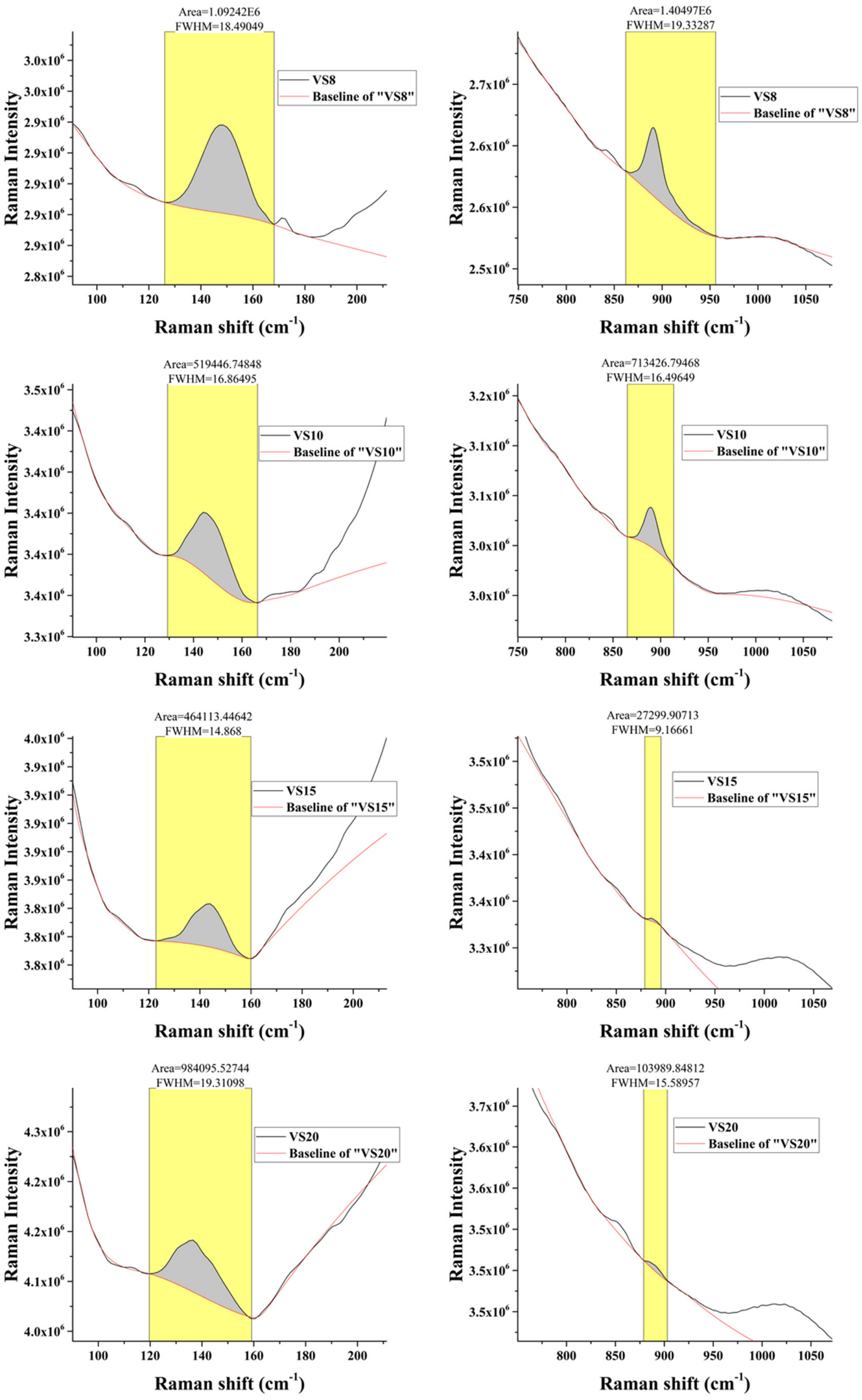

Figure S5. Detail of the Raman spectra of the studied samples. Area under the peaks at 145 and 890 cm^{-1} that corresponds to the VS8-VS20 samples.

Table S1. Area of the relevant peaks of all VS sample on the Raman spectra and their corresponding V_4O_9/V_2O_5 area ratio.

Sample	Determined area of the peak of ~145 cm^{-1} (V_2O_5)	Determined area of the peak of ~890 cm^{-1} (V_4O_9)	Ratio of the ratio of the area corresponding to 890 cm^{-1} to the area corresponding to 145 cm^{-1}
VS0			
VS1	3.14E+07	1.51E+05	0.005
VS2	1.78E+06	3.83E+05	0.215
VS4	6.00E+05	5.29E+05	0.882
VS6	1.28E+06	2.24E+06	1.754
VS8	1.09E+06	1.40E+06	1.286
VS10	5.19E+05	7.13E+05	1.373
VS15	4.64E+05	2.73E+04	0.059
VS20	9.84E+05	1.04E+05	0.106

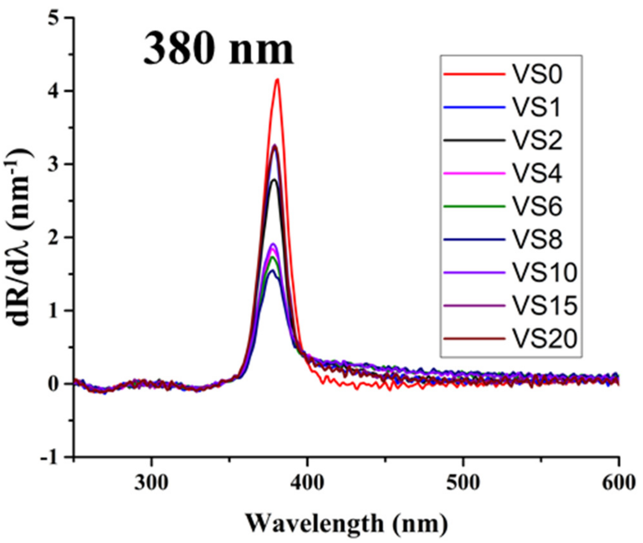

Figure S6. First-order derivative of the measured reflectance spectra for VS0–VS20 samples

.

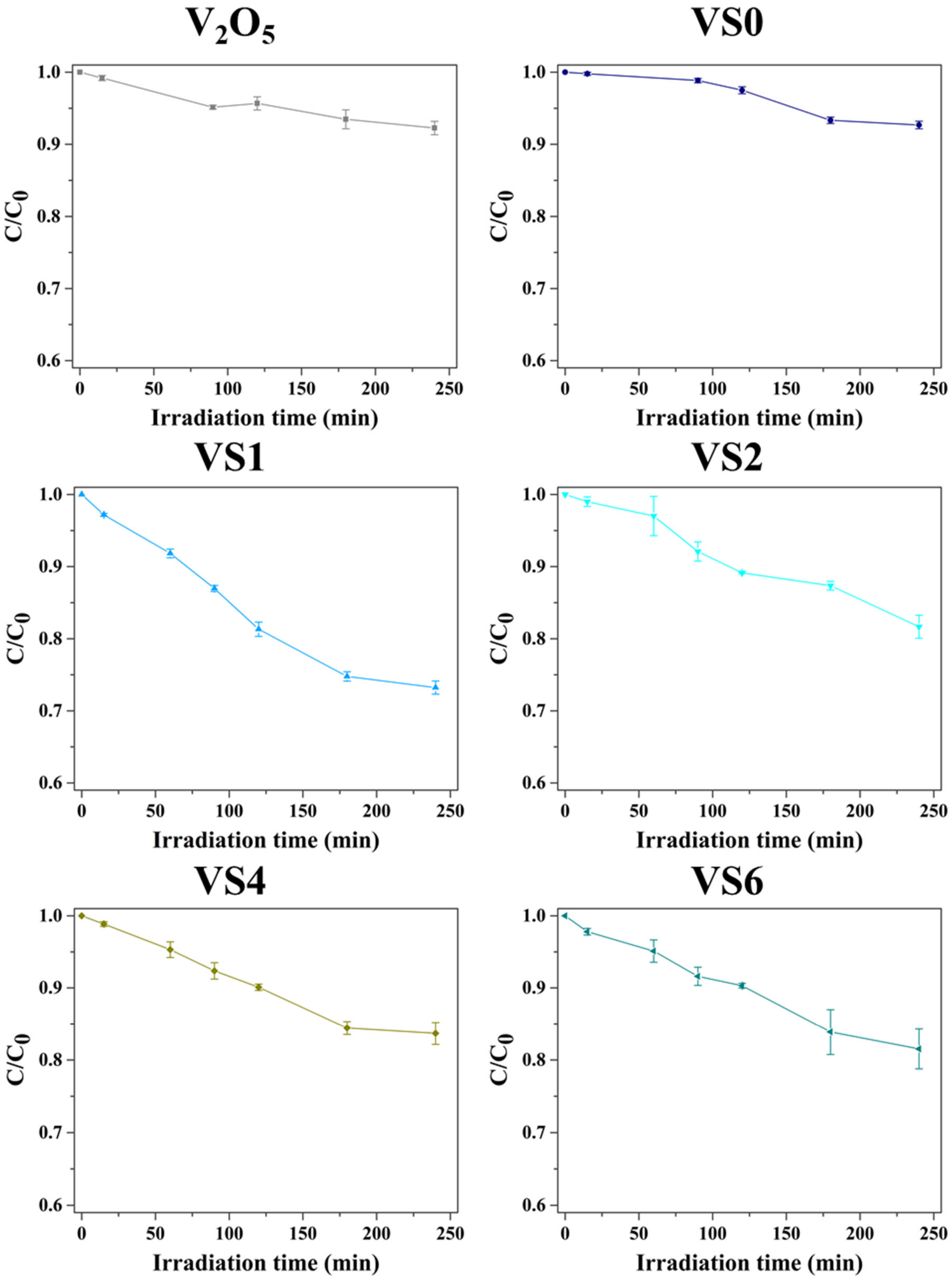

Figure S7. Average and standard deviation of repeated phenol conversion tests for V_2O_5 and VS0-VS6.

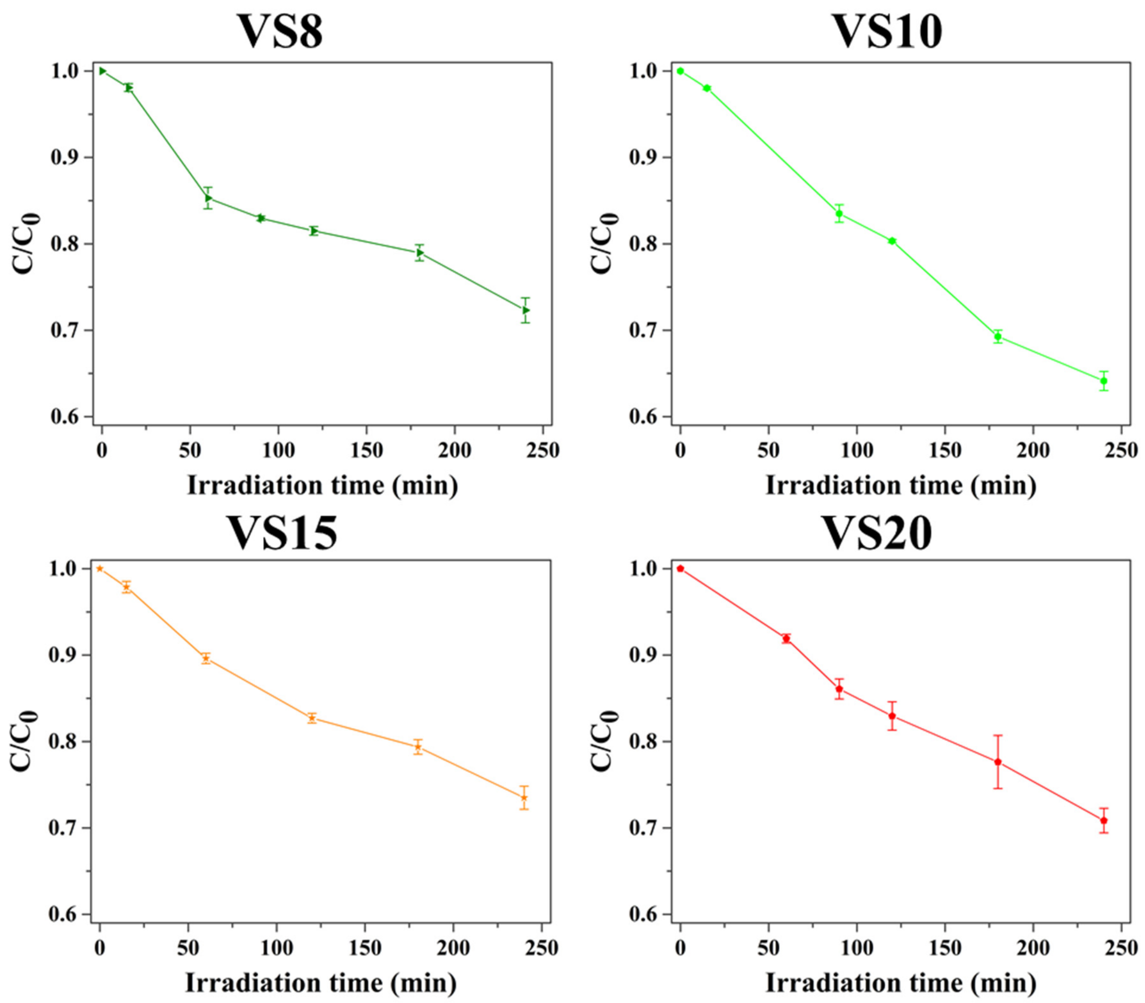

Figure S8. Average and standard deviation of repeated phenol conversion tests for V_2O_5 and VS8-VS20.

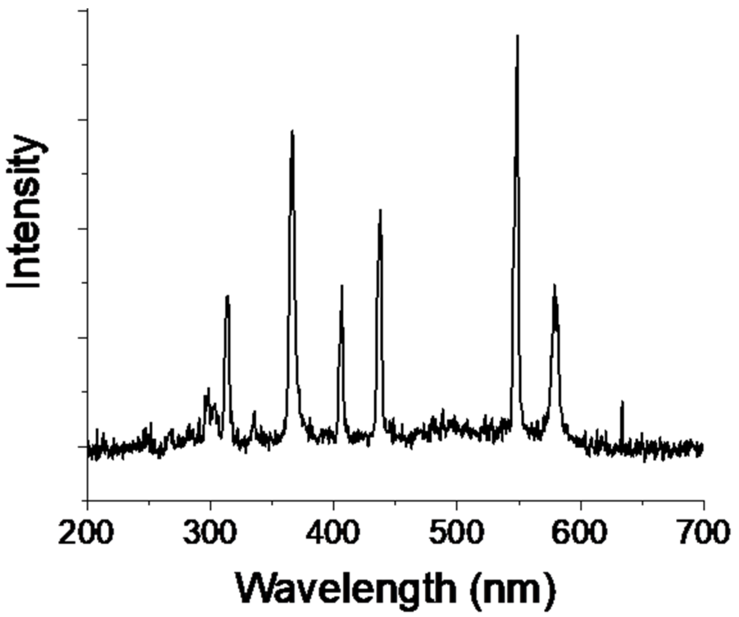

Figure S9. Emission spectrum of the light source used during the photocurrent density measurements.

Figures S10–S13 show the results of the thermogravimetric analysis of samples VS0, VS2, VS8, and VS20. In none of the cases did the weight loss reach 0.5%, and all curves look the same, with no trends apparent. The detectable weight loss (relative to the mass of the sample weighed) is below the resolution limit of the balance. This means that the sample exhibits a high degree of stability and that organic synthesis residues were successfully removed during the preliminary calcination.

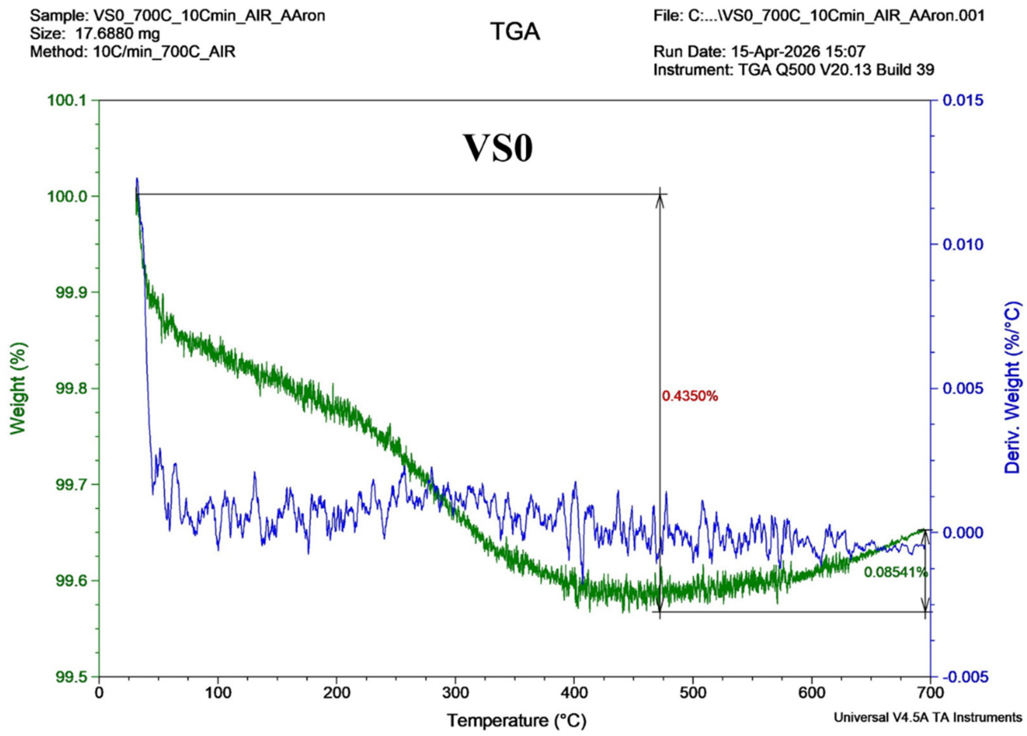

Figure S10. TG curve of VS0.

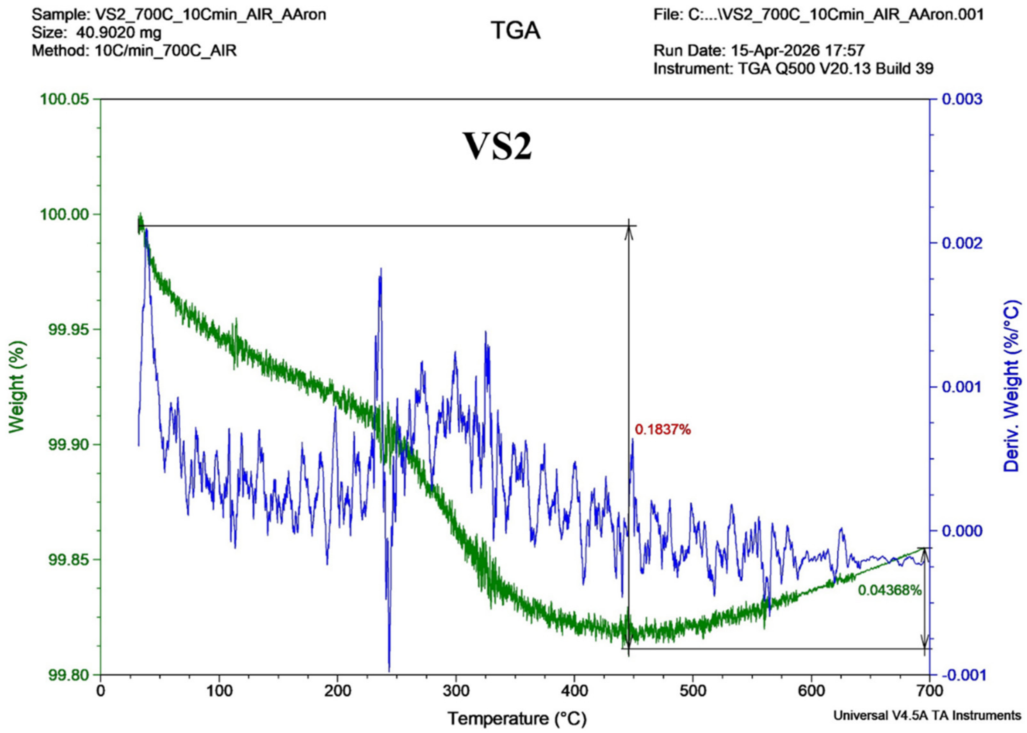

Figure S12. TG curve of VS2.

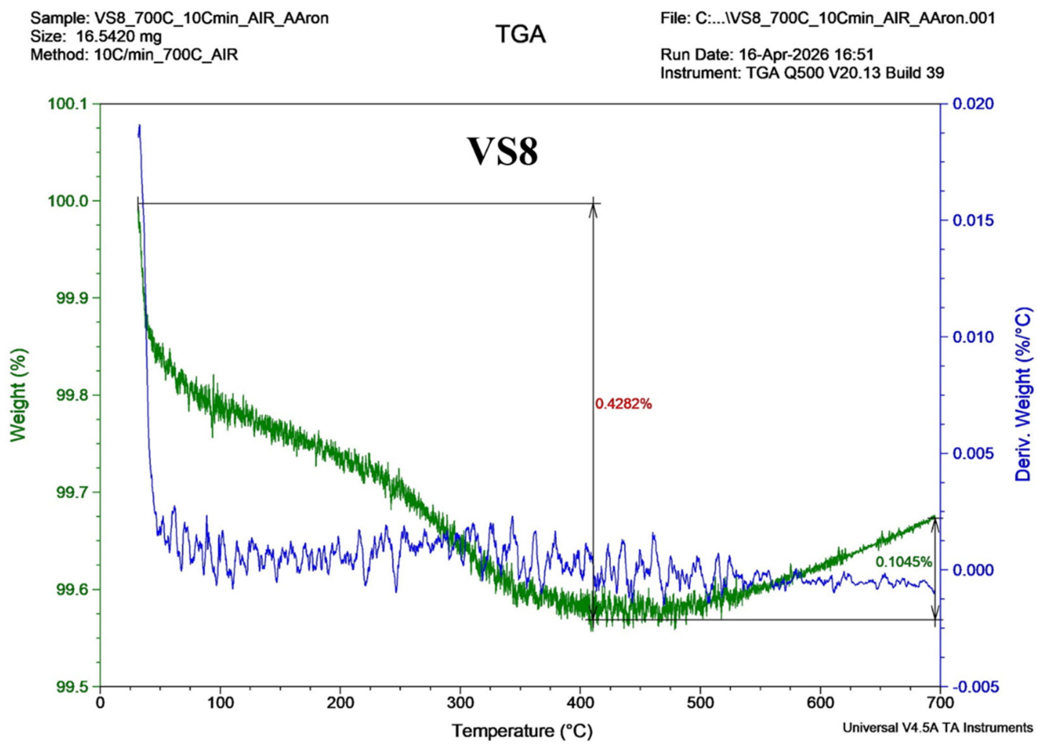

Figure S11. TG curve of VS8.

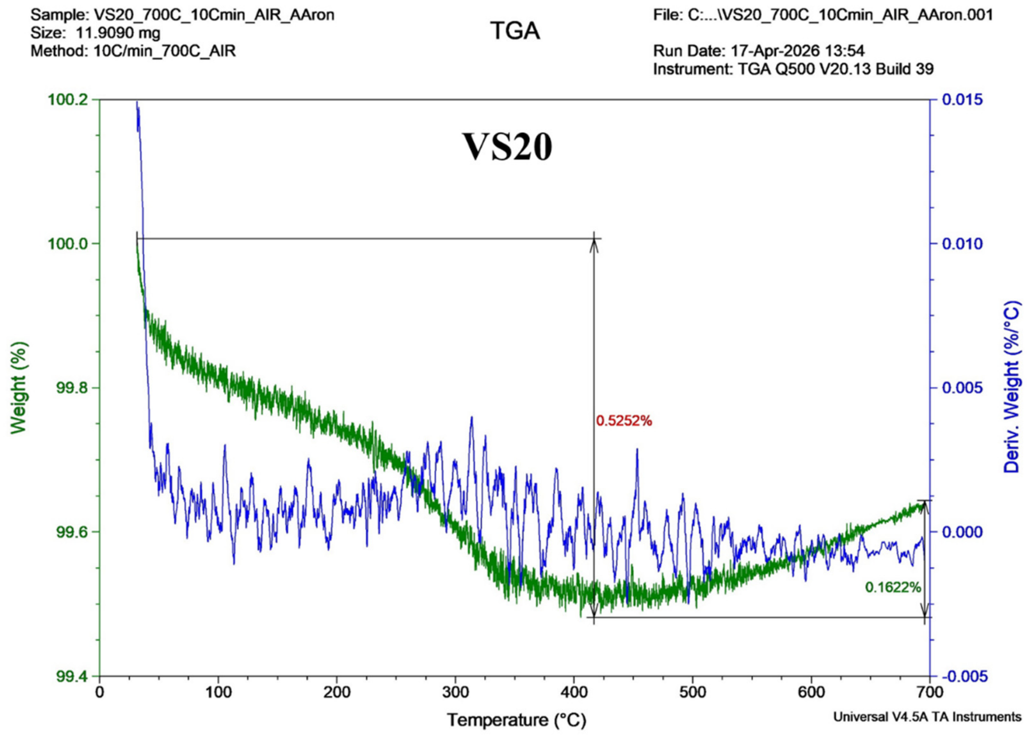

Figure S13. TG curve of VS20.

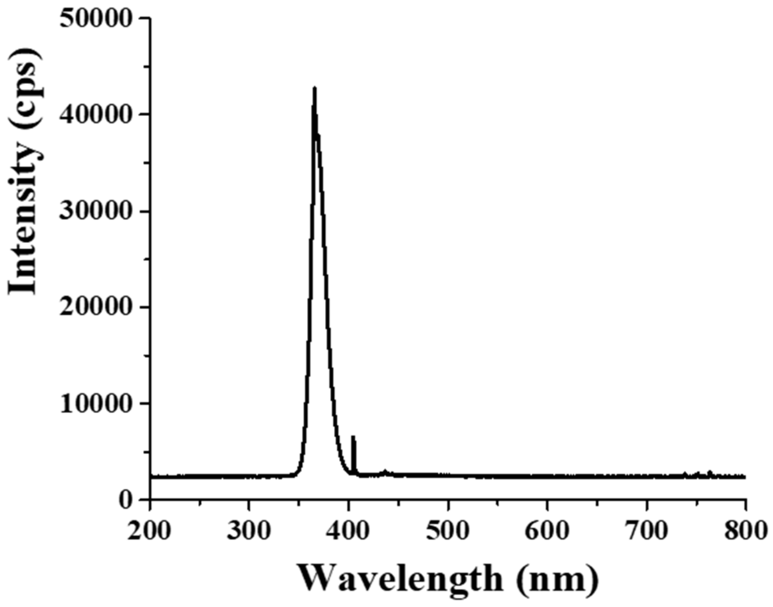

Figure S14. Emission spectrum of the light source used during photocatalytic experiments.

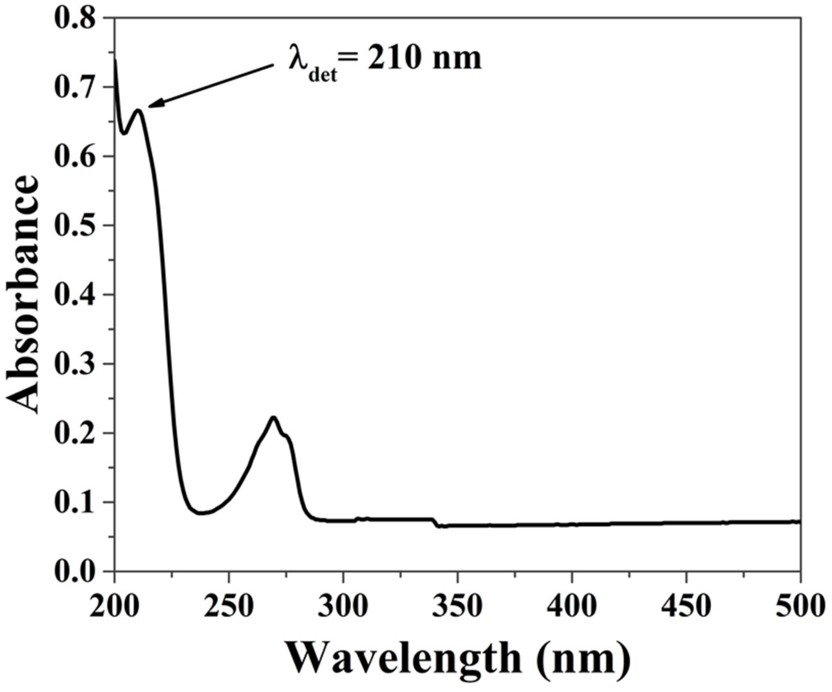

Figure S15. Absorbance spectrum of phenol.
